# Simultaneous dual-contrast multi-phase liver imaging using spectral photon-counting computed tomography: a proof-of-concept study

**DOI:** 10.1186/s41747-017-0030-5

**Published:** 2017-12-22

**Authors:** Daniela Muenzel, Heiner Daerr, Roland Proksa, Alexander A. Fingerle, Felix K. Kopp, Philippe Douek, Julia Herzen, Franz Pfeiffer, Ernst J. Rummeny, Peter B. Noël

**Affiliations:** 1 Department of Diagnostic and Interventional Radiology, Klinikum rechts der Isar, Technical University of Munich, Ismaningerstrasse 22, 81675 München, Germany; 2 Philips GmbH Innovative Technologies, Research Laboratories, Hamburg, Germany; 3 Department of Interventional Radiology and Cardio-vascular and Thoracic Diagnostic Imaging, Louis Pradel University Hospital, Bron, France; 4 Chair of Biomedical Physics, Department of Physics and School of BioEngineering, Technical University of Munich, Garching, Germany; 5 Institute for Advanced Study, Technical University of Munich, Garching, Germany

**Keywords:** Computed tomography (CT), Dual-contrast computed tomography, Gadolinium-based contrast agent, Gadolinium mapping, Iodine-based contrast agent, Iodine mapping, Liver, S*p*ectral photon-counting computed tomography (SPCCT)

## Abstract

**Background:**

To assess the feasibility of dual-contrast spectral photon-counting computed tomography (SPCCT) for liver imaging.

**Methods:**

We present an SPCCT in-silico study for simultaneous mapping of the complementary distribution in the liver of two contrast agents (CAs) subsequently intravenously injected: a gadolinium-based contrast agent and an iodine-based contrast agent. Four types of simulated liver lesions with a characteristic arterial and portal venous pattern (haemangioma, hepatocellular carcinoma, cyst, and metastasis) are presented. A material decomposition was performed to reconstruct quantitative iodine and gadolinium maps. Finally, a multi-dimensional classification algorithm for automatic lesion detection is presented.

**Results:**

Our simulations showed that with a single-scan SPCCT and an adapted contrast injection protocol, it was possible to reconstruct contrast-enhanced images of the liver with arterial distribution of the iodine-based CA and portal venous phase of the gadolinium-based CA. The characteristic patterns of contrast enhancement were visible in all liver lesions. The approach allowed for an automatic detection and classification of liver lesions using a multi-dimensional analysis.

**Conclusions:**

Dual-contrast SPCCT should be able to visualise the characteristic arterial and portal venous enhancement with a single scan, allowing for an automatic lesion detection and characterisation, with a reduced radiation exposure.

## Key points

- Based on our simulations, dual-contrast liver imaging with spectral photon counting computed tomography is feasible.
- A dual-contrast injection protocol (first gadolinium-based, second iodine-based) enables simultaneous multiphase scanning.
- The arterial contrast enhancement is visible thanks to the gadolinium-based contrast agent while the portal venous enhancement is visible thanks to the iodine-based contrast agent.
- Dual-contrast liver SPCCT imaging could help reducing radiation exposure.

## Background

Computed tomography (CT) is a standard imaging technique for detection and characterisation of focal liver lesions, the majority of them being benign (cysts and haemangiomas). In clinical routine it is important to differentiate benign lesions from malignant lesions, as the liver is a preferential organ for metastases of many primary tumours and benign and malignant lesions may coexist. Accurate diagnosis of hepatocellular carcinoma (HCC) is also important as it is the most common primary malignancy in the liver and the second leading cause of cancer-related mortality in the world [1–3].

With respect to oncological liver diagnostics, conventional contrast-enhanced CT is the workhorse in the clinical day-to-day routine. A controversial discussion concerns dynamic phases (native, early and late arterial, portal venous, equilibrium) in the setting of different liver pathologies [4–6]. Today, a standard CT liver protocol typically includes an arterial and portal venous contrast-enhanced series and, when thought to be necessary, a native unenhanced scan.

Detection and characterisation of liver lesions on conventional CT is based on differences in the attenuation values between lesions and the normal liver parenchyma. This difference occurs due to a tissue-dependent uptake of contrast agent (CA). In contrast to standard energy-integrating detectors, spectral photon-counting computed tomography (SPCCT) provides additional information on the energy spectrum of x-ray photons after passing through the patient and can thus detect spectral differences in the attenuation. This technology enables *material decomposition*, i.e. the analysis of different material components within the examined object.

The aim of our study was to explore the feasibility of a new approach for liver imaging using two CAs with different x-ray attenuation properties for an improved visualisation and automated detection and characterisation of lesions: a dual-contrast single-scan SPCCT protocol. More specifically, our aim was to investigate if this can be achieved by a specifically adjusted injection protocol, with arterial contrast distribution of one CA and portal venous enhancement of another CA. To achieve this goal, we present the potential of a sequential injection procedure, including a first intravenous injection of a gadolinium-based CA (CA1) and a second injection of an iodine-based CA (CA2), with a specific time gap depending on the individual circulation time. The scan acquisition is simulated at the time point of portal venous distribution for CA1 and at the time of arterial phase for CA2.

## Methods

This retrospective in-silico study was conducted in accordance to the guidelines of the local institutional review board. Anonymised CT datasets of one participant with a healthy liver (n = 1) and four patients, each of them with characteristic liver lesions (HCC, haemangioma, cyst, and metastasis) were used as a template for the numerical experiment. The diagnosis of liver lesions was confirmed by biopsy for HCC and metastasis while follow-up studies including magnetic resonance imaging confirmed the diagnosis of haemangioma and cyst.

### Dual-contrast injection protocol

**Fig. 1:**
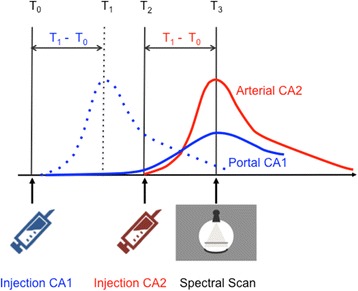
Injection protocol and timing characteristics for dual-contrast agent enhanced multi-phase SPCCT of the liver. The portal venous phase is reached within T_3_ (~70 s) following CA injection in humans, whereas the exact time point of arterial distribution (T_1_–T_0_) depends on the individual patient blood flow. For dual-contrast SPCCT imaging, synchronised portal venous distribution of CA1 and arterial distribution of CA2 at one time point is necessary (= T_3_). The sequence is started at T_0_, where CA1 is injected. At T_3_, CA1 shows a portal venous distribution. The time period T_1_–T_0_ defines the time necessary for enhancement in arterial phase. Then, CA2 is injected at T_2_ to assure arterial phase of CA2 at T_3_. At T_3_, the SPCCT scan is performed, with an arterial distribution of CA2 and a portal venous contrast of CA1. *Blue dotted line*, arterial distribution of CA1; *blue line*, portal venous distribution of CA1; *red line*, arterial distribution of CA2

For dual-contrast liver imaging, we defined a dedicated injection protocol (Fig. 1) with a sequential application of two different CAs. The attenuation curve of CA1 (gadolinium-based CA) within a region of interest (ROI) in the abdominal aorta was used as a test bolus to determine the individual patient-specific timing of the blood circulation and the maximum of arterial contrast enhancement. This information is essential to precisely forecast the time point of maximal arterial enhancement of the liver by CA2 (iodine-based CA).

At time point T_0_, CA1 (Magnograf®, Bayer Pharma AG, Berlin, Germany, administered at a dose of 0.2 mL/kg body weight, with an injection rate of 4 mL/s) is injected generating a maximal arterial enhancement of the liver at time point T_1_, followed by injection of CA2 at T_2_ (Iomeron 370, administered at a dose of 1 mL/kg body weight, with an injection rate of 4 mL/s). The time difference ΔT = T_1_ – T_0_ represents the period from CA injection to maximum of arterial enhancement. Sequential injection of CA1 and CA2 leads to a dual-phase dual-contrast distribution at the time point T_3_ = T_2_ + ΔT, with CA1 in the portal venous and CA2 in the arterial phase (see Fig. 1). At time point T_3_, the SPCCT examination is performed to simultaneously assess the contrast distribution of both CAs in the liver at different phases.

### Numerical SPCCT experiment

The numerical experiment was set up to be as realistic as possible by modelling both the energy-dependent attenuation characteristics of the patients and the actual physical performance of a SPCCT scanner. The number of detected photons in a detector pixel mainly depend on the three major components: the x-ray tube spectrum, the detector response and the attenuation properties of the patient [7]. In order to mimic an SPCCT acquisition, a realistic x-ray tube spectrum model [8] and a realistic detector response model (measurements at the European Synchrotron Radiation Facility [ESRF] in 2014, not published) were utilised. The resulting photon counts for each x-ray were calculated using the approach presented by Roessl and Proksa [9]. Poisson-distributed noise was added to the calculated photon counts. The simulation parameters match an already existing SPCCT prototype, where axial scans over 360° are obtained with a tube current of 50 mA, a tube voltage of 120 kVp, scanner rotation time of 1 s and 2400 projections per rotation. The noise threshold in the detector modelling was set to 30 keV and all images were reconstructed on a voxel grid of 0.39 × 0.39 × 0.25 mm [10].

### Preparation of numerical liver phantom and liver lesions

**Fig. 2:**
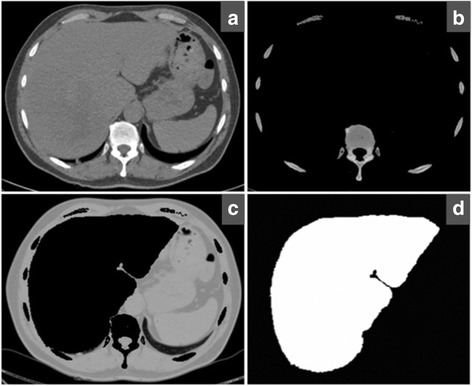
Preparation of the patient-inspired in-silico synthetic liver model for the numerical SPCCT experiments. **a** Original CT image of a healthy liver, (**b**) segmented bone, (**c**) segmented soft tissues, and (**d**) liver part of the original CT image. The liver is removed from the soft tissue image and is replaced in the synthetic liver phantom with a homogenous attenuation of 50 HU

To gain information of the attenuation characteristics in a real patient, we used an axial slice of a multi-phase liver CT scan and substituted the liver with a synthetic liver model. For this, we segmented the CT image by thresholding the data into a bone and soft tissue image (Fig. 2), followed by manual segmentation and replacement with a synthetic liver model with homogeneous CT values of 50 HU. A total of four common liver lesions have been selected from the clinic’s patient database by an experienced radiologist (D.M. nine years of experience).

**Fig. 3:**
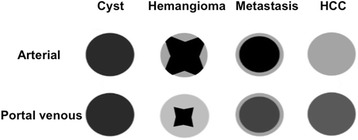
Selected shapes for the four liver lesions used in this study. The *grey values* indicate the distribution of the contrast uptake during the arterial and portal phase. *Light grey* stands for a high contrast uptake and *dark grey* for a low contrast uptake. The uptake of the HCC typically presents with a homogenous, high uptake during the arterial phase followed by washout in the portal phase. The haemangioma typically shows the ‘closing iris’ structure in the transition from arterial to portal venous distribution. The cyst does not uptake CA, in either the arterial or the portal venous phase. The metastasis has a small rim with a high CA uptake during both phases and a core with no enhancement during arterial phase and a low enhancement during the portal phase

Characteristics of arterial and portal venous contrast enhancement of these lesions were considered as follows (Fig. 3). The cyst does not enhance in both the arterial and the portal venous phase with a CT value of about 0 HU. HCC typically shows an arterial hyper-perfusion in the arterial contrast phase (+30 HU compared to liver enhancement) and a washout phenomenon in the venous phase (– 15 HU compared to liver enhancement). Haemangioma typically presents with a ‘closing iris’ pattern of enhancement, with a peripheral nodular hyper-vascularisation in the arterial phase (+75 HU compared to liver enhancement) and a centripetal contrast filling in the venous phases (+55 HU compared to liver enhancement). The metastasis (typically from colorectal cancer) shows a peripheral rim-like enhancement in the arterial phase (100 HU), which is further increased in the portal venous phase images (120 HU). The core of the metastasis was simulated without enhancing in arterial phase images and a little uptake in portal venous phase (35 HU). The liver lesions were added into the homogenous synthetic liver model, each with three different sizes: 5 mm, 10 mm and 20 mm, respectively. The 20 mm and 10 mm liver lesions were positioned in the right lobe of the liver in segment VIII and segment VII and the 5 mm lesions had their position in segment II according to Couinaud’s system of liver anatomy [11]. The enhancement of healthy liver was 60 HU in the arterial and 90 HU in the portal venous phase.

### Material decomposition

We employed a projection-based maximum-likelihood method for the material decomposition into water, CA1, and CA2 [7]. This method uses the photon x-ray spectrum, the linear attenuation coefficients of water, CA1, and CA2 together with the spectral detector response function [7, 9]. The decomposition algorithm estimates the material composition that fits best to the simulated noisy photon counts for each projection and detector pixel. More precisely, the multi-bin data were pre-processed and an integrated conventional CT image was obtained. After further corrections, a maximum likelihood material decomposition of the attenuation into a water, iodine, and gadolinium material basis was performed (see below).

To test the feasibility of spectral CT liver imaging with respect to its clinical applicability, we carefully adjusted the dose to be comparable to that used in conventional CT liver imaging protocols. Therefore, a conventional CT simulation using an energy-integrating detector of the chosen dataset was carried out to determine the nominal x-ray dose yielding an image noise of approximately 20 HU in the liver using the same reconstruction parameters as for the SPCCT simulation [10].

### Image reconstruction and processing

The outcome of the material decomposition are three material projection datasets (CA1, CA2, and water). The anti-correlated noise in the material images is suppressed by an iterative image based statistical de-noising algorithm [11]. Pixel-by-pixel based image analyses (e.g. cluster analysis or support vector machine) can be applied to the final material images to make best use of the simultaneously acquired arterial and portal venous phases. The three materials CA1, CA2, and water can be understood as a three-dimensional (3D) vector space. Each point $\overrightarrow{x}$ in this space represents a different combination of water, CA1, and CA2. Image pixels belonging to the same tissue type form clusters in the 3D vector space. In case the clusters do not or only partially overlap, the different types of tissue can be identified. We model a cluster for tissue type *t* (e.g. HCC, metastasis…) by a 3D joint real normal distribution, as follows:

$$ {p}^t\left(\overrightarrow{x}\right)={\prod}_{i=\left\{ water, CA1, CA2\right\}}\frac{1}{\sqrt{2\pi }{\sigma}_i^t}\mathit{\exp}\left(-\frac{1}{2}{\left(\frac{x_i-{\mu}_i^t}{\sigma_i^t}\right)}^2\right), $$

where: *x*_*i*_ with *i* = {*water*, *CA*1, *CA*2} are the entries of $\overrightarrow{x}$ describing a point in the 3D vector space; ${\mu}_i^t$ with *i* = {*water*, *CA*1, *CA*2} are the coordinates of the centre of the cluster for tissue type *t*; and ${\sigma}_i^t$ with *i* = {*water*, *CA*1, *CA*2} are the standard deviations of the cluster along the material axes.

Correlated noise between the material images was suppressed to a level allowing to neglect it in ${p}^t\left(\overrightarrow{x}\right)$. The value ${p}^t\left(\overrightarrow{x}\right)$ describes the likelihood for a point $\overrightarrow{x}$ to belong to the tissue type *t*. Thus, displaying ${p}^t\left(\overrightarrow{x}\right)$ for each image pixel is called likelihood map for tissue type *t*. The scatter plot and the likelihood representation of the decomposed material data were used to proof the lesion detection capability.

## Results

**Fig. 4:**
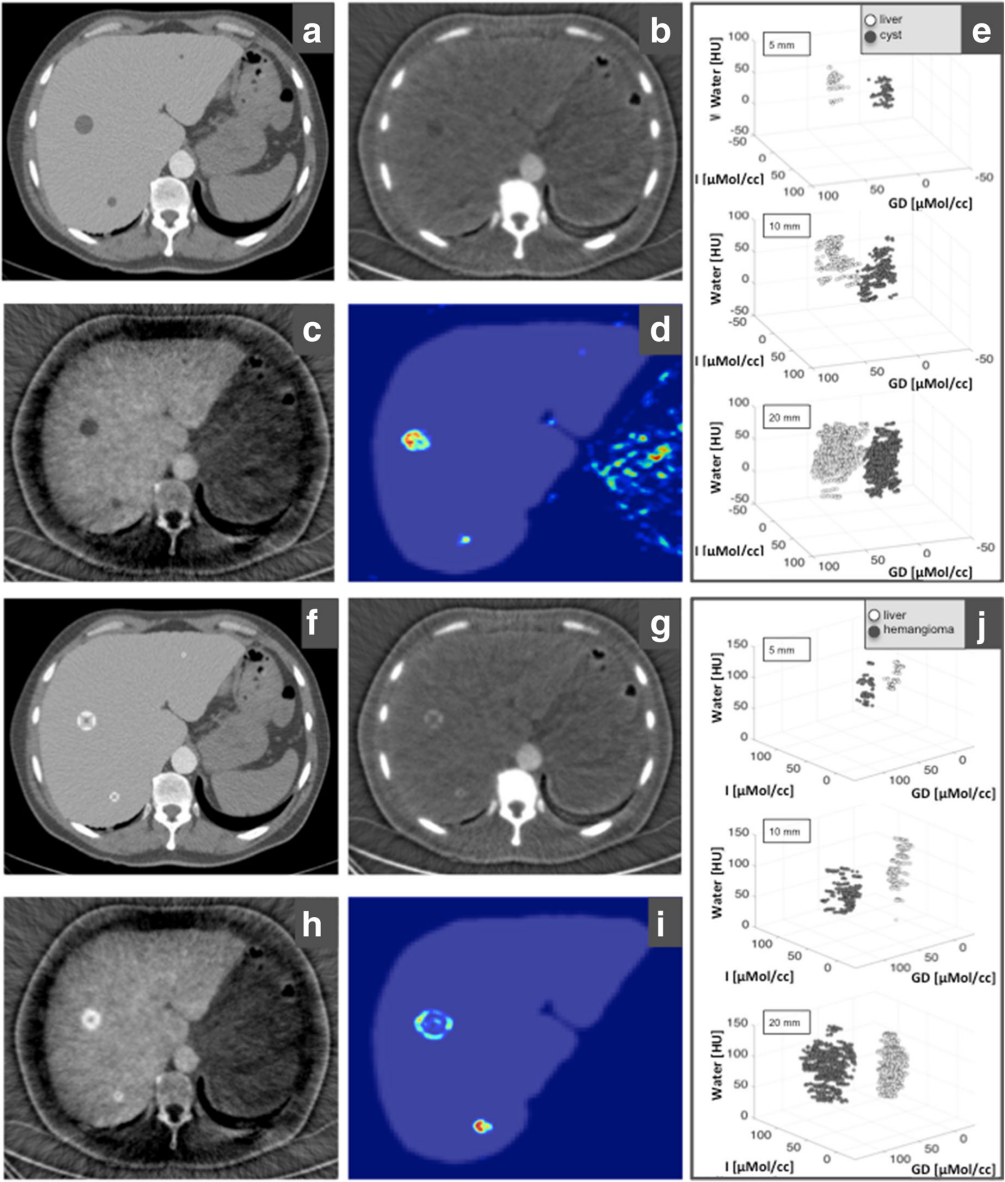
SPCCT imaging of benign liver lesions, exemplified by cysts (**a**–**e**) and haemangiomas (**f**–**j**). **a**, **f** Conventional CT image. **b** Iodine-enhanced image, showing cysts without any contrast uptake in the arterial phase. **c** Gadolinium-enhanced image showing cysts in portal venous phase. **g**, **h** Iodine- and gadolinium-enhanced image of haemangiomas, with a typical closing-iris pattern. **d** Likelihood map showing the 20-mm, 10-mm, and 5-mm diameter cysts. **i** For haemangiomas, the smallest lesion was missed in the calculated likelihood map. **e**, **j** *Scatter plots* illustrate the results of integrative analysis of SPCCT images with regard to the content of iodine and gadolinium as well as the non-contrast fraction (= water) for all lesions and all sizes without significant overlap of liver parenchyma (*light grey*) and lesion (*dark grey*). For this, two ROIs in the three material images were evaluated; one ROI was placed in the lesion (*dark grey markers*) and the other in healthy liver tissue (*light grey markers*). Image windowing: **a** and **f**, level/window 50/300 HU; **b** and **g**, level/window 25/100 μmol/cc of iodined CA; **c** and **h**, level/window 25/100 μmol/cc of gadolinium-based CA

**Fig. 5:**
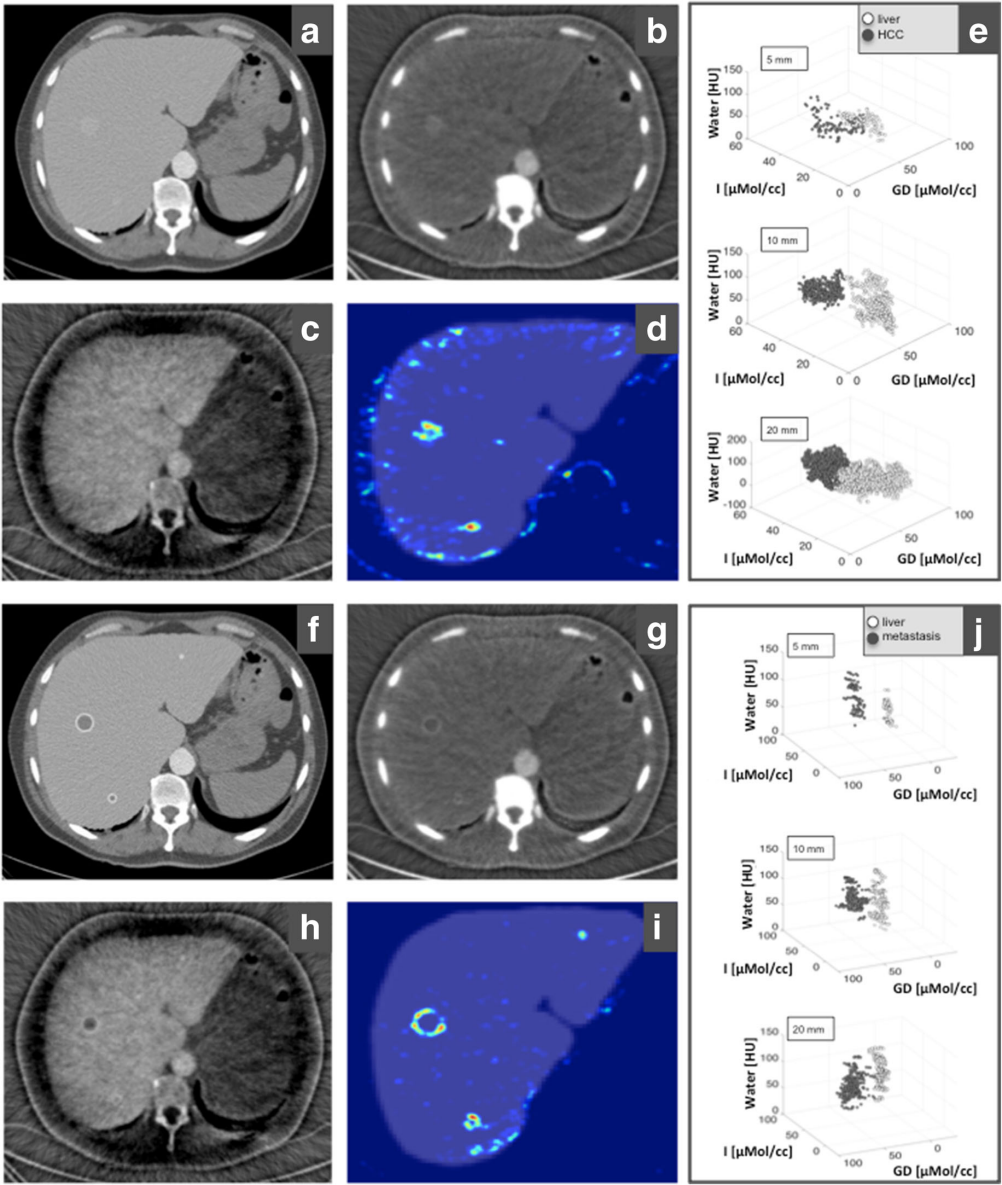
SPCCT imaging of malignant liver lesions, exemplified by HCC (**a**–**e**) and metastasis (**f**–**j**). **a**, **f** Conventional CT image. **b** Iodine-based image, showing HCC with a typical hyper-enhancement in arterial phase. **c** Gadolinium-based image of HCC in portal venous phase, which shows only subtle early washout with hypodense presentation in the largest lesion. **g**, **h** Iodine- and gadolinium-based image of metastases, with a typical ring-like enhancement in the arterial phase (**g**), increased in the portal venous phase (**h**). **d** *Likelihood map* for HCC, which shows HCC in the right lobe but misses the small lesion in the left lobe, due to artifacts along the liver contour. **i** For metastases, the likelihood map clearly illustrated all the three lesions. **e**, **j** The *scatter plots* show an overlap for the fractionised analysis for iodine, gadolinium and water for HCC (**e**, *dark grey*), whereas all metastases (**j**, *dark grey*) are clearly separated from liver parenchyma (**e**, **j**, *light grey*). For this, two ROIs in the three material images were evaluated, one placed in the lesion (*dark grey markers*) and the other one in healthy liver tissue (*light grey markers*). Image windowing: **a** and **f**, level/window 50/300 HU; **b** and **g**, level/window 25/100 μmol/cc of iodined CA; **c** and **h**, level/window 25/100 μmol/cc of gadolinium-based CA

The material decomposition was allowed to differentiate between liver enhancement of CA1 and CA2, as shown in Figs. 4 and 5, where we show the distribution of both CAs within the liver parenchyma, in addition to a conventional CT image, for comparison. The iodine images present with high concentration values in the aorta and no visible contrast enhancement of the liver parenchyma, as it is typical for the arterial phase. In the gadolinium images, the portal venous distribution of CA1 results in a homogenous enhancement of the liver.

Cysts and haemangiomas, two typical benign liver lesions, usually present with a characteristic behaviour in arterial and portal venous phases. Fig. 4 outlines the results for these benign conditions. Cysts appear with lower concentration values compared to normal liver parenchyma in the iodine (Fig. 4b) and gadolinium (Fig. 4b) maps, indicating that there is no uptake of CA1 and CA2 in the arterial and portal venous phases. The likelihood map illustrates the cystic lesions of all sizes within the normal liver. Two separate clusters can be identified in the scatter plot for cysts and for liver tissue for all sizes of liver lesions (Fig. 4e).

SPCCT images of haemangiomas reveal the typical peripheral nodal enhancement in the arterial phase, followed by a centripetal contrast filling of the lesion in portal venous phase (Fig. 4). All three lesions are visible in standard CT, iodine, and gadolinium images. However, the likelihood map for haemangiomas does not indicate the 5-mm lesion in the left lobe of the liver. Scatter diagrams of haemangiomas and normal liver showed no overlap for all sizes, indicating an excellent discrimination of liver lesions.

Figure 5 summarises the results of SPCCT for two common malignant liver lesions, HCC (Fig. 5a–e) and peripheral hyper-vascular metastases (Fig. 5f–j). SPCCT illustrates arterial hyper-enhancement by CA2, followed by washout with a hypodense presentation compared to normal liver tissue in the portal phase images for 20-mm and 10-mm lesions. The 5-mm lesion, however, can only be identified on the attenuation CT and the iodine image in the arterial phase. Scatter plots show two distinct clusters for HCC and for liver tissue for the lesion sizes of 20 mm and 10 mm. For the smallest lesions both cluster were positioned close by, resulting in a slight overlap of SPCCT imaging features of HCC and normal liver (Fig. 5e).

The simulated metastases with a ring-like enhancement in the arterial phase, and particularly in the portal venous phase, were clearly visible in all images, including the likelihood map, for all sizes of lesions. In addition, the corresponding scatter plots illustrate an excellent delineation of metastases and healthy liver parenchyma, without relevant overlap between the imaging features in both tissues.

## Discussion

In this study we present our first results of an in-silico simulation of simultaneous dual-contrast multi-phase SPCCT liver imaging. A dedicated perfusion protocol was defined to simultaneously assess the arterial and portal venous distribution of two CAs. We evaluated the typical presentation of the arterial and portal venous contrast enhancement for four characteristic types of liver lesions. Further, the likelihood maps, based on the information of both CAs in different dynamic phases, illustrated areas with high probability for the respective lesion within normal liver tissue. This new dual-contrast SPCCT protocol therefore shows a potential for a clinically relevant simultaneous assessment of the arterial and portal venous contrast enhancement of liver lesions at one time point using a single SPCCT scan acquisition, together with a virtually unenhanced image. This approach goes beyond the currently possible ability of dual-energy systems, which can be used to acquire simultaneous iodine and gadolinium images [12], but not a third additional virtual unenhanced image simultaneously.

In SPCCT, the x-ray photon is directly converted into an electrical pulse signal, whose amplitude is indicative for the energy of the initial x-ray photon [13–17]. Without considering K-edge absorption, the energy-dependent attenuation of the human body can be modelled as a linear combination of a set of materials [18] and is then typically expressed in a basis decomposition of two materials or, in terms of the underlying physical interaction processes, photo-electric effect and Compton-scattering. If K-edges are taken into account, some materials feature an abrupt increase of the attenuation due to resonant ionisation of a K-shell electron at a certain energy (K-edge) within the energy range relevant for imaging, roughly from 40 keV up to 120 keV. This characteristic attenuation behaviour at the K-edge can be used to perform a specific material decomposition with a high signal-to-noise ratio. Gadolinium features a K-edge at 50.24 keV and thus is an ideal candidate for K-edge CT imaging, even better than iodine, which is the standard CA for contrast-enhanced CT scanning with a relatively low K-edge energy at 33.17 keV. SPCCT allows for a valid differentiation between iodine and gadolinium, even if both iodine-based and gadolinium-based contrast CAs can have the same density values on standard CT [10].

In SPCCT, material decomposition delivers a set of basis images, which typically features higher noise than an integrated image. But the noise in the decomposed images is anti-correlated so that, using recent reconstruction methods, this noise can efficiently be reduced [19]. Such de-noising methods not only lower the noise, but also yield a higher contrast resolution.

Notably, liver parenchyma receives a dual blood supply from the portal vein and hepatic artery. Normal liver parenchyma is mainly perfused via the portal vein, whereas HCCs are mostly supplied with blood from the hepatic artery. Therefore, information of perfusion patterns has an important impact on diagnostic decision-making [20]. Thus, a dedicated liver protocol includes at least one arterial and one portal venous contrast phases. Individual criteria can also require a native unenhanced scan, e.g. for the detection of calcifications and in patients with a history of transarterial chemoembolisation with iodined oil [21]. According to the Dose Index Registry Report from the American College of Radiology, the median radiation dose in terms of dose-length product for a standard single CT acquisition of the abdomen is 445 mGy × cm [22]. As a consequence, triple scan examination of the liver leads to a radiation dose of around 1335 mGy × cm. Dual-contrast SPCCT of the liver offers detailed three-phase contrast information with radiation exposure similar to that needed for a single CT scan of the abdomen.

With standard single-energy CT, a differentiation between normal liver parenchyma and focal liver lesions is achieved by comparing the changes in contrast enhancement between arterial and portal venous phase. In the past, poorly aligned multi-phase CT examination protocols made it difficult to assess these changes in contrast enhancement of the same anatomic areas and post-processing techniques (including motion-correction of repetitive CT scans) were essential for reliable results of extracted perfusion parameters [23, 24]. Using the method presented here, all dynamic information is extracted from a single SPCCT scan and hence is inherently co-registered (on a voxel-by-voxel basis), circumventing any previous issues due to subsequent multi-phase scans or motion artifacts (e.g. those determined by different depths of breathing). This further allows for a pixel-by-pixel evaluation of contrast enhancement of a lesion, as advanced analysis using scatter plots and likelihood maps require a perfect spatial match of the vector space components. This perfect match can be provided by the protocol here proposed.

Of note, we have used a gadolinium-based agent as first CA and an iodine-based agent as second CA. In future studies it will be necessary to address the question if this order is the preferred one or if iodine-based agent as first CA and gadolinium-based agent as second CA might be advantageous.

One future perspective of our approach could be adding a delayed dynamic phase, a clinically relevant issue particularly for characterising haemangioma and HCC [25]. While technologically feasible, such an approach would require a third CA. This, however, is presently a challenge, as iodine-based CAs and gadolinium-based CAs are the only available CAs with a potential for this use.

There are limitations of our study. First, we used numerical experiments to evaluate the imaging features in dual-contrast liver SPCCT. Although the simulations were performed using sophisticated tools and incorporated most physical effects in the SPCCT system, the findings have to be proved in an in-vivo study with a real SPCCT system in future. Second, there is currently very little clinical experience with the subsequent injection of iodine-based CA and gadolinium-based CA in humans. Therefore, pharmacological testing of potential adverse side effects will be necessary before an application of our presented contrast injection protocol can be used in patients.

Third, there is a general limitation with respect to the minimum lesion size assessable with this approach, as the contrast uptake and characteristic patterns cannot be studied when the lesion becomes too small. Finally, we should consider pharmacodynamic considerations about the long-term deposition of gadolinium in patients, which presently is a subject to intense discussions in the community.

In conclusion, we proved the concept of single-scan dual-CA CT as a new approach for K-edge SPCCT imaging, which exploits the spectral information for simultaneously assessing the gadolinium-based and iodine-based liver enhancement in different dynamic phases. Our simulation results have shown that we can successfully detect and visually discriminate the clinical presentation of four typical liver lesions by simultaneously evaluating unenhanced, arterial, and portal venous phases at one time point by a single scan, with a potential for dose reduction in clinical practice.

## Abbreviations

3D: Three-dimensional
CA: Contrast agent
CT: Computed tomography
HCC: Hepatocellular carcinoma
ROI: Region of interest
SPCCT: Spectral photon-counting computed tomography
